# Testosterone replacement therapy is associated with increased odds of Achilles tendon injury and subsequent surgery: a matched retrospective analysis

**DOI:** 10.1186/s13047-023-00678-0

**Published:** 2023-11-11

**Authors:** J. Alex Albright, Mary Lou, Elliott Rebello, Jonathan Ge, Edward J. Testa, Alan H. Daniels, Michel Arcand

**Affiliations:** 1 Warren Alpert Medical School of Brown University, Providence, RI 02903 USA; 2 Department of Orthopaedics, Brown University Warren Alpert Medical School, Providence, RI 02912 USA

**Keywords:** Achilles, Testosterone, Tendon Injury, Tendinopathy, Surgery

## Abstract

**Background:**

Prescription of testosterone replacement therapy (TRT) has increased in the United States in recent years, and though anabolic steroids have been associated with tendon rupture, there is a paucity of literature evaluating the risk of Achilles tendon injury with TRT. This study aims to evaluate the associative relationship between consistent TRT, Achilles tendon injury, and subsequent surgery.

**Methods:**

This is a one-to-one matched retrospective cohort study utilizing the PearlDiver database. Records were queried for patients aged 35–75 who were prescribed at least 3 consecutive months of TRT between January 1, 2010 and December 31, 2019. Achilles tendon injuries and subsequent surgeries were identified using ICD-9, ICD-10, and CPT billing codes. Multivariable logistic regression was used to compare odds of Achilles tendon injury, Achilles tendon surgery, and revision surgery, with a *p*-value < 0.05 representing statistical significance.

**Results:**

A sample of 423,278 patients who filled a TRT prescription for a minimum of 3 consecutive months was analyzed. The 2-year incidence of Achilles tendon injury was 377.8 (95% CI, 364.8–391.0) per 100,000 person-years in the TRT cohort, compared to 245.8 (95% CI, 235.4–256.6) in the control (*p* < 0.001). The adjusted analysis demonstrated TRT to be associated with a significantly increased likelihood of being diagnosed with Achilles tendon injury (aOR = 1.24, 95% CI, 1.15–1.33, *p* < 0.001). Of those diagnosed with Achilles tendon injury, 287/3,198 (9.0%) of the TRT cohort subsequently underwent surgery for their injury, compared to 134/2,081 (6.4%) in the control cohort (aOR = 1.54, 95% CI, 1.19–1.99, *p* < 0.001).

**Conclusions:**

There is a significant association between Achilles tendon injury and prescription TRT, with a concomitantly increased rate of undergoing surgical management. These results provide insight into the risk profile of TRT and further research into the science of tendon pathology in the setting of TRT is an area of continued interest.

**Supplementary Information:**

The online version contains supplementary material available at 10.1186/s13047-023-00678-0.

## Background

 Achilles tendon injuries are common musculoskeletal pathologies that are characterized by pain and swelling about the heel and decreased athletic performance and mobility [1]. While prevalent among athletes, the condition also considerably affects the general population [2, 3]. Risk factors for Achilles tendon injury include excessive mechanical overload and training errors, along with factors such as increased age, hypertension, obesity, diabetes, inflammatory arthropathies, and corticosteroid use [4]. Non-surgical management is generally pursued first and has reasonably high success rates. This involves a tailored combination of rest, orthotics, non-steroidal anti-inflammatory drugs, and eccentric exercise. However, for unknown reasons, nearly a third of patients continue to have persistent symptoms despite undergoing conservative care and proceed to undergo surgery [5].

Low levels of serum sex hormones can have detrimental impacts on patient’s health, with presentation including sexual dysfunction and decreases in muscle mass, strength, bone density, libido, and concentration [6, 7]. In patients with persistently low hormone concentration, testosterone replacement therapy (TRT) can be prescribed to restore physiologic levels and treat the symptoms of testosterone insufficiency [8]. Rate of TRT prescription increased more than threefold in the United States from 2001 to 2011, with most patients having no clear indication for therapy [9].

In addition to the well-understood risk of cardiovascular disease with the use of TRT, hepatic dysfunction, infertility, and tendon pathologies have also been associated with TRT [10]. Specifically, androgenic-anabolic steroids (AASs) have been associated with tendon rupture. Two hypotheses are proposed in the literature: AAS-induced rapid muscle hypertrophy is not accompanied by corresponding tendon adaptation and causes injury or AASs directly damage the structure of tendons, thereby leading to injury or rupture [10, 11]. In rodents, AASs have demonstrated negative effects on collagen synthesis, a critical aspect of tendon maintenance and repair, and in a cross-sectional cohort study, tendon rupture in AAS users was found to be 9 times more likely than in nonusers [12, 13]. Additionally, in a recently published study, TRT has been associated with increased rates of primary rotator cuff tears and secondary, ipsilateral cuff tears, making the relationship between TRT and Achilles tendon injury of significant interest [14]. Thus, this study aimed to determine the risk of Achilles tendon injury and subsequent surgery in patients who had received TRT. Our hypothesis was that patients who had previously been prescribed exogenous testosterone would have an increased likelihood of Achilles tendon injury and subsequently need increased rates of surgical management in comparison to controls, with these associations most notable among the older adult populations and similar between men and women.

## Methods

### Data source

This retrospective comparative analysis was performed using the de-identified data from M157Ortho dataset within the larger PearlDiver database (PearlDiver Technologies, Colorado Springs, CO). This database is generated using insurance claims from Humana Inc. and contains longitudinal data from over 150 million patients from January 2010 through December 2021, and has been an established resource used for orthopedic surgery research questions that require large datasets to answer [15]. These data allow researchers to characterize and analyze short-, medium-, and long-term rates and trends of various conditions, postoperative complications, and sequalae using the International Classifications of Disease, ninth revision (ICD-9) and tenth revision (ICD-10) and Current Procedural Terminology (CPT) codes. This dataset also includes prescription medication information, including the duration and time of prescriptions filled. For the current study, these data were used to calculate the rates of Achilles tendon injury and subsequent surgery in patients who filled prescriptions of exogenous testosterone for at least three consecutive months and compare it to a control [14].

### Creating the cohorts

The testosterone group was generated by querying the M157Ortho dataset for patients matching the inclusion criteria, and then subsequently filtering out patients based on exclusion criteria. Inclusion criteria for the testosterone group were patients aged 35 to 75 who filled a prescription for exogenous testosterone for a minimum of 3 consecutive months with a minimum of two years follow up within the time period. The time range restriction allowed for a minimum of 2 years of follow-up for each patient. Exclusion criteria were patients with a diagnosis of rheumatologic disease, cancer, or mitochondrial disease, and those who were not active within the dataset (i.e., they dropped their coverage or changed insurance) within the two years after their prescription was filled, which facilitated a more accurate calculation of rate. The control cohort was created by first generating a random sample of patients between the ages of 35 and 75 who had never filled a prescription for testosterone before, and then utilizing one-to-one exact matching to yield a final cohort of patients matched with the testosterone cohort on age, sex, Charlson comorbidity index, and a diagnosis of tobacco use or diabetes. All codes used can be referenced in the Supplemental Table.

### Identifying injuries and surgery

The rate and incidence (per 100,000 person-years) of index Achilles tendon injury were calculated over a two-year period following 3 consecutive months of testosterone and compared to the control cohort over the same time period. This analysis was performed for the combined cohort, as well as sex- (male or female) and age-specific (35 to 45, 46 to 55, 56 to 65, and 66 to 75 years) cohorts. These injuries were identified using ICD-9 and ICD-10 diagnosis codes. The primary Achilles tendon surgery rate was calculated by tracking the initial Achilles tendon injury diagnosis and determining the number of patients who filed a claim with an associated CPT code for surgery of the Achilles tendon. The rate of Achilles surgery was used as a proxy for the severity of the initially identified injury as more severe cases, including complete Achilles tendon rupture, are more likely to undergo surgery [16].

### Statistical analysis

The incidence of index Achilles tendon injury was calculated for all cohorts and compared using the exact Poisson method. Chi-square tests were used for bivariate comparisons between the two matched cohorts. Multivariable logistic regression was used to compare the two cohorts while adjusting for other specific comorbidities, including a diagnosis of hypogonadism, chronic kidney disease, osteoporosis, overweight/obesity (body mass index > 25), class III obesity (body mass index > 40), alcohol use, osteoarthritis, lung disease, congestive heart failure, and dementia. These variables were included in the regression model to adjust for other factors that may influence either the rate of initial injury or rate of undergoing surgical management (i.e., poor surgical candidate secondary to concomitant lung or heart disease) [17–20]. The number needed to harm, a metric used to quantify the number of patients needed to be treated to cause harm to one patient, was calculated for the overall cohort by dividing 1 by the absolute risk increase. The absolute risk increase is the absolute difference in the testosterone event rate and the control event rate. Adjusted odds ratios (aOR) and their respective 95% confidence intervals were generated and reported for each comparison. To protect patient identity, cohorts comprised of less than 11 patients are reported as “-1” by PearlDiver and thus were reported as “< 11” throughout this manuscript. Statistical analyses are still able to be performed on these smaller cohorts, but the specific value is simply not reported. A *p*-value of < 0.05 was determined to represent statistical significance *a priori*. All statistical analyses were performed using the R Statistical Package (v4.2.1; R Core Team 2022, Vienna, Austria) embedded within PearlDiver.

## Results

**Fig. 1:**
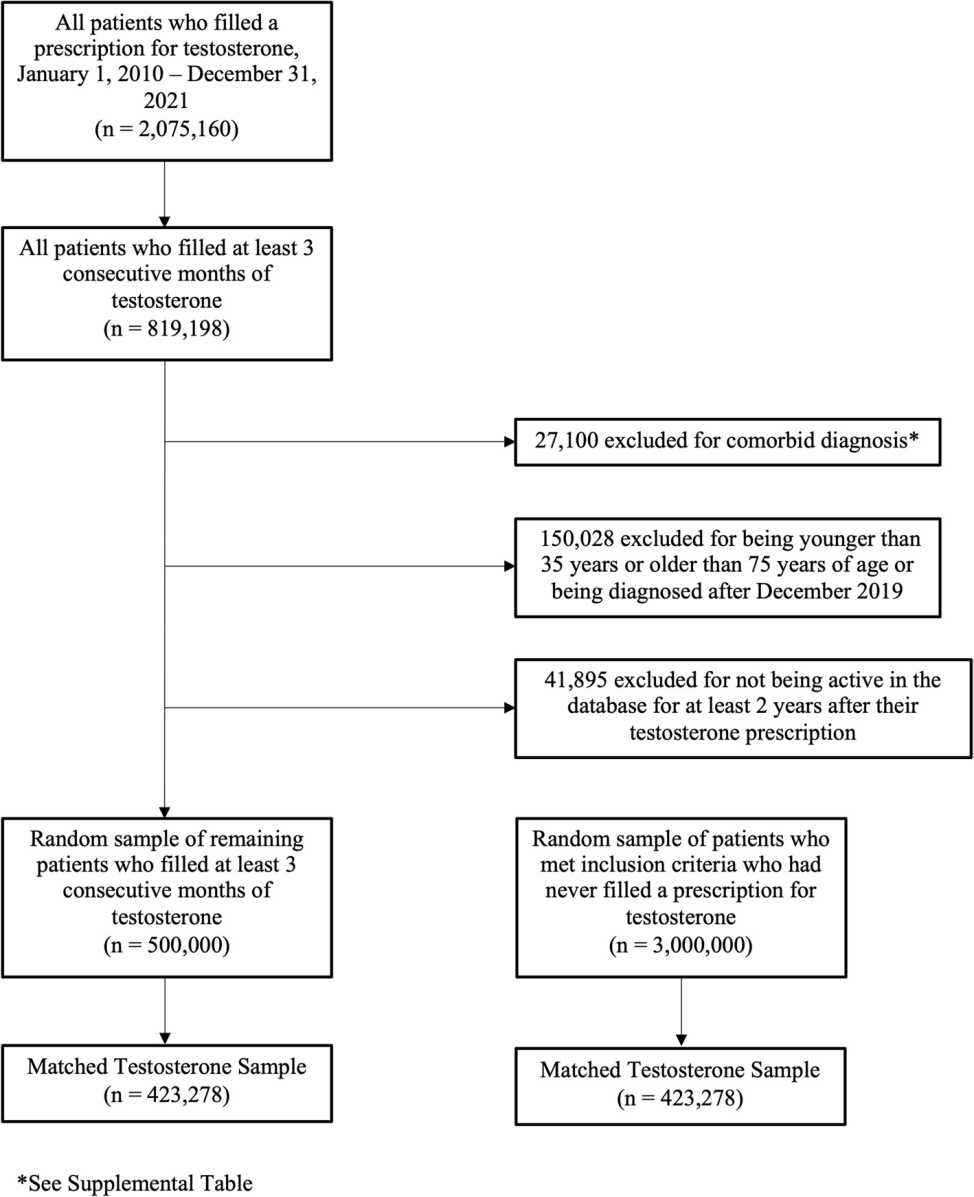
Flow diagram depicting inclusion and exclusion criteria and the number of patients excluded at each point

A total of 2,075,160 patients filled a prescription for exogenous testosterone, with 819,198 of those patients filling the prescription for a minimum of 3 months. Following exclusions and the 1:1 exact matching process, 423,278 patients comprised both the testosterone and control cohorts (Fig. 1). Prior to matching, both cohorts had significant demographic differences, including prior tobacco use and diabetes (Table 1). However, following the matching process, these differences no longer persisted. Table 2 depicts the number of patients in each of the age- and sex-specific cohorts.

**Table 1:** Demographics comparison of the unmatched and matched cohorts

Characteristic	Unmatched Cohorts			Matched Cohorts		
	Prior Testosterone Use	Control	*p*-value	Prior Testosterone Use	Control	*p*-value
Sex, n male (%)	421,362 (84.3)	1,222,831 (40.8)	**< 0.001**	345,369 (81.6)	345,369 (81.6)	1.000
Age, mean ± SD	56.3 ± 11.7	55.4 ± 10.5	**< 0.001**	54.8 ± 10.4	54.8 ± 10.4	1.000
CCI, mean ± SD	0.8 ± 1.5	0.1 ± 0.5	**< 0.001**	0.4 ± 0.8	0.4 ± 0.8	1.000
Tobacco Use, n (%)	164,212 (32.8)	872,296 (29.1)	**< 0.001**	130,130 (30.7)	130,130 (30.7)	1.000
Diabetes, n (%)	203,436 (40.7)	1,106,875 (36.9)	**< 0.001**	157,449 (37.2)	157,449 (37.2)	1.000

Bold indicates statistical significance

*CCI* Charlson comorbidity index, *SD* Standard deviation

**Table 2:** A breakdown of the number of patients in both the experimental and control cohort following the matching process

Population		Testosterone Use, n (%)	Control Cohort, n (%)
Sex	Age		
All Patients		423,278 (100.0)	423,278 (100.0)
Male		345,369 (81.6)	345,369 (81.6)
	35 to 45	80,608 (19.0)	80,608 (19.0)
	46 to 55	103,754 (24.5)	103,754 (24.5)
	56 to 65	92,961 (22.0)	92,961 (22.0)
	66 to 75	68,046 (16.1)	68,046 (16.1)
Female		77,909 (18.4)	77,909 (18.4)
	35 to 45	10,120 (2.4)	10,120 (2.4)
	46 to 55	28,320 (6.7)	28,320 (6.7)
	56 to 65	28,185 (6.7)	28,185 (6.7)
	66 to 75	11,284 (2.7)	11,284 (2.7)

Over the two-year follow-up period, the incidence of Achilles tendinopathy was 377.8 (95% CI, 364.8–391.0) injuries per 100,000 person-years, compared to 245.8 (95% CI, 235.4–256.6) injuries per 100,000 person-years (*p* < 0.001). The adjusted multivariable regression demonstrated that patients who previously filled prescriptions for testosterone were significantly more likely to be diagnosed with Achilles tendon injuries when compared to the control (aOR = 1.24, 95% CI, 1.15–1.33, *p* < 0.001), with an overall number needed to harm of 371. For male patients, there was a significantly increased likelihood of developing an Achilles tendon injury in each age cohort. Female patients had a significantly increased likelihood in all age cohorts except the 35 to 45 cohort. The two sex- and age-specific cohorts with the greatest increase in adjusted odds were the males aged 66 to 75 (aOR = 1.66, 95% CI, 1.35–2.03, *p* < 0.001) and the females aged 66 to 75 (aOR = 1.82, 95% CI, 1.21–2.75, *p* = 0.004). See Table 3 for all unadjusted and adjusted comparisons.

**Table 3:** Comparison of the likelihood of being diagnosed with Achilles tendon injury over a two-year period

Population and Time Period of Interest	Number of Injuries, n (%)		Regression Analysis	
	Testosterone Use	Control	aOR^a^ (95% CI)	*p* - value
All Patients	3,198 (0.76)	2,081 (0.49)	1.24 (1.15 - 1.33)	**< 0.001**
Male	2,565 (0.74)	1,630 (0.47)	1.38 (1.29 - 1.49)	**< 0.001**
35 to 45	651 (0.81)	441 (0.55)	1.40 (1.21 - 1.61)	**< 0.001**
46 to 55	846 (0.82)	567 (0.55)	1.28 (1.13 - 1.46)	**< 0.001**
56 to 65	710 (0.76)	435 (0.47)	1.51 (1.31 - 1.74)	**< 0.001**
66 to 75	358 (0.53)	187 (0.27)	1.66 (1.35 - 2.03)	**< 0.001**
Female	633 (0.81)	451 (0.58)	1.44 (1.27 - 1.64)	**< 0.001**
35 to 45	74 (0.73)	58 (0.57)	1.29 (0.89 - 1.87)	0.175
46 to 55	241 (0.85)	185 (0.65)	1.33 (1.08 - 1.64)	**0.007**
56 to 65	244 (0.87)	167 (0.59)	1.54 (1.25 - 1.90)	**< 0.001**
66 to 75	74 (0.66)	41 (0.36)	1.82 (1.21 - 2.75)	**0.004**

Bold indicated statistical significance (*p* < 0.05)

*aOR* Adjusted odds ratio, *CI* Confidence interval

^a^adjusted used multivariable logistic regression to additionally control for hypogonadism, chronic kidney disease, osteoporosis, overweight/obesity (body mass index > 25), class III obesity (body mass index > 40), alcohol use, osteoarthritis, lung disease, congestive heart failure, and dementia

The testosterone cohort was also more likely to undergo surgery on their Achilles tendon injury when compared to the control cohort (Table 4.) Of the patients diagnosed with Achilles tendon injury, 287/3,198 (9.0%) of the testosterone cohort subsequently underwent surgery for their injury, compared to just 134/2,081 (6.4%) in the control cohort (aOR = 1.54, 95% CI, 1.19–1.99, *p* < 0.001). The two sex- and age-specific cohorts with the greatest increase in odds were the males aged 56 to 65 (aOR = 2.07, 95% CI, 1.17–3.75, *p* = 0.014) and the females aged 56 to 65 (aOR = 3.13, 95% CI, 1.33–8.25, *p* = 0.013).

**Table 4:** Comparison of the likelihood of subsequently undergoing surgery for an Achilles tendon injury

Population and Time Period of Interest	Number of Repairs, n (%)		Regression Analysis	
	Testosterone Use	Control	aOR^a^ (95% CI)	*p* - value
All Patients	287 (9.0)	134 (6.4)	1.54 (1.19 - 1.99)	**0.001**
Male	231 (9.0)	115 (7.1)	1.24 (0.95 - 1.64)	0.118
35 to 45	67 (10.3)	47 (10.7)	1.00 (0.62 - 1.62)	0.989
46 to 55	71 (8.4)	36 (6.3)	1.23 (0.74 - 2.02)	0.421
56 to 65	68 (9.6)	21 (4.8)	2.07 (1.17 - 3.75)	**0.014**
66 to 75	25 (7.0)	11 (5.9)	1.08 (0.45 - 2.62)	0.863
Female	56 (8.8)	19 (4.2)	2.57 (1.50 - 4.55)	**< 0.001**
35 to 45	< 11 (N/A)	< 11 (N/A)	1.89 (0.24 - 17.85)	0.545
46 to 55	21 (8.7)	< 11 (N/A)	2.25 (0.95 - 5.77)	0.074
56 to 65	24 (9.8)	< 11 (N/A)	3.13 (1.33 - 8.25)	**0.013**
66 to 75	< 11 (N/A)	< 11 (N/A)	2.11 (0.35 - 17.55)	0.432

Bold indicated statistical significance (*p* < 0.05)

*aOR* Adjusted odds ratio, *CI* Confidence interval

^a^adjusted used multivariable logistic regression to additionally control for hypogonadism, chronic kidney disease, osteoporosis, overweight/obesity (body mass index > 25), class III obesity (body mass index > 40), alcohol use, osteoarthritis, lung disease, congestive heart failure, and dementia

## Discussion

The purpose of the current study was to evaluate the relationship between exogenous testosterone administration and Achilles tendon injury. Our results demonstrate that, when compared to a matched control cohort, the rate of Achilles tendon injury and subsequent surgery was significantly higher in patients prescribed TRT. Furthermore, this trend was found in both males and females even after statistical control for several medical comorbidities, most notably hypogonadism. These results demonstrate an important correlation between testosterone use and Achilles tendon injury and point to future clinical implications of testosterone prescription and usage. Much of prior literature has focused on the effects of anabolic steroids on tendon structure and health, but this study is the first to retrospectively analyze rates of Achilles tendon injuries and subsequent surgery in patients prescribed TRT.

In the Achilles tendon, testosterone has been linked to increased tendon stiffness, a reduced relaxin response, and inhibited matrix metalloproteinase (MMPs) activity, all of which negatively affect tendon integrity after repeated strain [21, 22]. At a molecular level, tendon health is maintained by a balance of MMPs and tissue inhibitors of matrix metalloproteinases (TIMPs) that is crucial for tissue healing, and the use of exogenous steroids has been demonstrated to significantly alter this balance, leading to collagen dysplasia in rats and a consequent increase in Achilles tendon injury [23–25]. In multiple studies on human AAS users, increased rates of tendon rupture were discovered compared to non-AAS users [12, 13, 26]. Multiple case reports on rare tendon avulsions and ruptures have also linked AAS usage of the patients to the cause of the tendon injury [27–29]. In the context of testosterone’s biochemical effects on tendon health and structure and its rising usage and prescription, it imperative to better understand the musculoskeletal complications associated with exogenous testosterone in order to minimize patient harm.

This study reports an increased odds of 1.38 in Achilles tendon injury in males and an increased odds of 1.44 in females with a minimum of three consecutive months of prescription TRT. While the current body of literature regarding prescription TRT and musculotendinous injury is scarce, a recent study by Testa et al. in a similar analysis demonstrated a significant increase in rotator cuff tears and revision rotator cuff repair [14]. A second similar study published by Smith et al. evaluated rates of rotator cuff repair (RCR) in patients that were sex-hormone deficient and they found an 89% increase in rates of RCR in males who were testosterone deficient and a 48% increase in estrogen-deficient females [30]. Although Smith et al. do not account for testosterone supplementation in sex-hormone-deficient patients, their study does allude to the complex relationship between sex-hormone derangements and musculotendinous health, which is in accordance with the present study. The present study builds on this literature by adjusting for previously diagnosed hypogonadism and other comorbidities associated with musculoskeletal injury when evaluating and comparing rates of Achilles tendon injury and subsequent surgery in patients prescribed consistent TRT, identifying increased odds of Achilles tendon injury and subsequent surgery regardless of hypogonadism status and after controlling for other comorbidities.

Prior studies observed increased rates of injury in male patients compared to female patients. Testa et al. found increased odds of rotator cuff tears in both male and female patients prescribed TRT, with males having a higher aOR of 4.73 in comparison to 3.49 for females [14]. In another study on the association of TRT with rates of distal biceps tendon injury (BTI), Rebello et al. demonstrated that male patients prescribed TRT were 4.68 times more likely to experience distal BTI, while females prescribed TRT were 1.82 times more likely [31]. There are many speculative explanations for these findings, including the prevalence weight training, strain from higher force loads across the tendon, or rates of aerobic exercise participation [32–35]. Our study observed increased odds of general Achilles tendon injury in both men and women prescribed TRT, but in contrast to the above studies, the association was similar between men and women. However, when only considering full-thickness Achilles tendon ruptures, previous literature has identified a 5:1 ratio of men to women in the occurrence of rupture, which may be more consistent with other published literature on tendon injuries and TRT [32]. Additionally, other studies have demonstrated the decreased stiffness of the Achilles tendon in women, including Intziegianni et al. who measured an increased tendon cross-sectional area deformation under a lower applied force in female tendons, pointing to a more compliant and less injury-prone tendon that could be associated with the decreased rates of Achilles tendon rupture in women [32, 33]. Nevertheless, it remains difficult to truly explain this association without further understanding of the interplay between testosterone and estrogen in maintaining tendon health and as the effects of estrogen on tendon size, stiffness, and collagen content are still debated, this interconnection is likely multifaceted and remains a topic for additional research [36–38].

Within both male and female cohorts in this study, the highest adjusted odds were seen in the aged 66 to 75 cohorts. As hypogonadism increases with age, the presence of this trend in the oldest age cohort regardless of patient hypogonadism status indicates that there is likely a separate explanation for this observation. One such reason is that tendon physiology and structure can change significantly due to aging. In a study by Pardes et al., Achilles tendon properties were measured and compared among rats of various ages, with increased passive stiffness and decreased range of motion seen in older rats. Older rats were also found to have Achilles tendons with lower maximum stresses and lower elasticities compared to those of younger rats, additional properties that make a tendon more susceptible to injury [39]. Aging has a similar impact on tendons in humans, and causes additional biological changes such as decreased cellularity, reduced proliferation and activity of tenocytes, and decreased organization of collagen fibers that all weaken tendon structure [40]. The association of TRT with decreased obesity, improved energy levels, and increased self-esteem in patients with hypogonadism implicates a possibility for increased physical activity in these patients as well, which, in combination with the biomechanical and physiologic changes in tendon structure, could explain the increased odds of Achilles tendon injury in the oldest cohorts [41]. The interplay between degenerative tendon changes and participation in physical activity of each age group could also determine the acuity of the Achilles tendon injury sustained, thereby affecting the odds of undergoing surgery.

The decision to undergo surgery is also heavily impacted by the physician’s evaluation of a patient’s healing potential, which decreases with age [40]. Thus, despite having the highest odds of Achilles tendon injury, the aged 66 to 75 TRT cohorts did not have significant increases in likelihood of surgery possibly due to physician preference of nonsurgical treatment for older patients regardless of TRT prescription. Among the remaining age-sex TRT cohorts, both males and females aged 56 to 65 had significant odds of undergoing surgery for their Achilles tendon injury, which, speculatively, may be a result of an increase in participation in high-intensity exercise along with less physician preference for nonsurgical treatment out of concern for age-related complications of surgery in comparison to the oldest cohorts [42]. As this trend is not present for the male cohorts aged 56–65, in which the odds were significantly higher than the control cohorts, there likely is an additional layer of complexity in the relationship among sex, comorbidities, and TRT prescription that could explain the increased odds of surgical intervention for Achilles tendon injury for specific age-sex cohorts.

### Limitations

As with all retrospective administrative claims database studies, this study is not without limitations. As a retrospective and observational study, the results of this study cannot be used to make any causal inference between TRT and Achilles tendon injury. In addition, while this study does use one-to-one matching on patient demographics and the Charlson comorbidity index and subsequently controls for several comorbid medical conditions in the multivariable analyses, there are several other factors that place patients at increased risk of injury that could not be captured in this study, such as patient baseline activity level. Similarly, this study could not control for the specific Achilles tendon injury each patient incurred; there is no specific CPT code for Achilles tendon rupture and therefore we could not directly look at these injuries, which is an important entity to be considered more closely in future studies. We were unable to address additional informative factors including degree of injury, muscle atrophy, and degree of muscle retraction in potential ruptures, as well as details regarding the surgery, including specific method used, surgeon experience, or the location of the operation. We are likewise unable to determine the specific dosing of TRT, route of administration, changes in dosing over time, compliance with the prescribed dosing, or the testosterone levels of the patient over time. Therefore, this study is unable to draw any conclusions on the dose-related effect of TRT or the effect of route of administration on Achilles tendon injury and is only able to determine that there is an association between a minimum of three months of testosterone replacement therapy and increased rates of Achilles tendon injury. Finally, while this study does capture over 400,000 patients who filled prescriptions for TRT, this study only includes data from a single insurer, Humana Inc., and may not be representative of the greater national population that would include patients insured through other private companies, Medicare, or Medicaid. Notably, the much smaller sample sizes of female cohorts make it difficult to obtain a comprehensive perspective on the female population that is prescribed TRT, limiting insight into the sex differences in TRT- associated Achilles tendon injury.

## Conclusion

By using a national administrative claims database, this study observed a significant increase in the likelihood of both Achilles tendon injury and Achilles tendon injury requiring surgery in patients who filled a minimum of three consecutive months of prescription TRT. While prior studies have documented musculotendinous injury in the context of exogenous steroid administration, this is the first study to specifically associate prescription TRT with Achilles tendon injury and subsequent surgery. These results are important for the prescribing physicians and patients considering TRT to recognize when formulating treatment plans and are a valuable addition to the growing body of literature regarding the effects of testosterone therapy and musculoskeletal health.

### Supplementary Information

**Additional file** **1:** **Supplemental Table.** All International Classifications of Disease (ICD), ninth and tenth revision and Current Procedural Terminology (CPT) codes used.

## Data Availability

The data that support the findings of this study are available from PearlDiver, Inc. but restrictions apply to the availability of these data, which were used under license for the current study, and so are not publicly available. However, data are available from the authors upon reasonable request and with permission of PearlDiver, Inc.

## Abbreviations

TRT: Testosterone replacement therapy
OR: Odds ratio
aOR: Adjusted odds ratio
CI: Confidence interval
AAS: Androgenic-anabolic steroids
MMP: Matrix metalloproteinases
TIMP: Tissue inhibitors of matrix metalloproteinases
RCR: Rotator cuff repair
