# Supplementary material for: Testosterone replacement therapy is associated with increased odds of Achilles tendon injury and subsequent surgery: a matched retrospective analysis
Source: J Foot Ankle Res. 2023 Nov 11;16:76. doi: 10.1186/s13047-023-00678-0 (PMC10638827; doi:10.1186/s13047-023-00678-0)
Supplement: Supplementary file 1 — Additional file 1: Supplemental Table. All International Classifications of Disease (ICD), ninth and tenth revision and Current Procedural Terminology (CPT) codes used. [file 13047_2023_678_MOESM1_ESM.docx]

**Supplemental Table**: All International Classifications of Disease (ICD), ninth and tenth revision and Current Procedural Terminology (CPT) codes used

| Diagnosis | | Code(s) | | | | | | |
| --- | --- | --- | --- | --- | --- | --- | --- | --- |
|  | Achilles Tendon Injury | ICD-9-D-72671, ICD-9-D-72767, ICD-9-D-84509, ICD-10-D-M7661, ICD-10-D-M7662, ICD-10-D-M7660, ICD-10-D-S86001A, ICD-10-D-S86001D, ICD-10-D-S86001S, ICD-10-D-S86002A, ICD-10-D-S86002D, ICD-10-D-S86002S, ICD-10-D-S86009A, ICD-10-D-S86009D, ICD-10-D-S86009S, ICD-10-D-S86011A, ICD-10-D-S86011D, ICD-10-D-S86011S, ICD-10-D-S86012A, ICD-10-D-S86012D, ICD-10-D-S86012S, ICD-10-D-S86019A, ICD-10-D-S86019D, ICD-10-D-S86019S, ICD-10-D-S86021A, ICD-10-D-S86021D, ICD-10-D-S86021S, ICD-10-D-S86022A, ICD-10-D-S86022D, ICD-10-D-S86022S, ICD-10-D-S86029A, ICD-10-D-S86029D, ICD-10-D-S86029S, ICD-10-D-S86091A, ICD-10-D-S86091D, ICD-10-D-S86091S, ICD-10-D-S86092A, ICD-10-D-S86092D, ICD-10-D-S86092S, ICD-10-D-S86099A, ICD-10-D-S86099D, ICD-10-D-S86099S | | | | | | |
|  |  |  |  |  |  |  |  |  |
|  |  |  |  |  |  |  |  |  |
|  |  |  |  |  |  |  |  |  |
|  |  |  |  |  |  |  |  |  |
|  |  |  |  |  |  |  |  |  |
|  |  |  |  |  |  |  |  |  |
|  |  |  |  |  |  |  |  |  |
|  |  |  |  |  |  |  |  |  |
|  |  |  |  |  |  |  |  |  |
|  |  |  |  |  |  |  |  |  |
|  | Achilles Tendon Surgery | CPT-27650, CPT-27652, CPT-27654 |  |  |  |  |  | |
| Pharmacotherapy | |  |  |  |  |  | |  |
|  | Testosterone Replacement Therapy | DRUG-4-DIHYDROTESTOSTERONE, DRUG-DEPO-TESTOSTERONE, DRUG-ESTROGEN-METHYLTESTOSTERONE, DRUG-ESTROGEN_&_METHYLTESTOSTERONE, DRUG-FIRST-TESTOSTERONE, DRUG-FIRST-TESTOSTERONE_MC, DRUG-METHYLTESTOSTERONE, DRUG-METHYLTESTOSTERONE_MICRONIZED, DRUG-TESTOSTERONE, DRUG-TESTOSTERONE_CYPIONATE, DRUG-TESTOSTERONE_CYPIONATE_MICRO, DRUG-TESTOSTERONE_ENANTHATE, DRUG-TESTOSTERONE_MICRONIZED, DRUG-TESTOSTERONE_PROPIONATE | | | | | | |
|  |  |  |  |  |  |  |  |  |
|  |  |  |  |  |  |  |  |  |
|  |  |  |  |  |  |  |  |  |
|  |  |  |  |  |  |  |  |  |
|  |  |  |  |  |  |  |  |  |
|  |  |  |  |  |  |  |  |  |
|  |  |  |  |  |  |  |  |  |
| Exclusion Criteria | |  |  |  |  |  | |  |
|  | Marfan Syndrome | ICD-9-D-75982, ICD-10-D-Q8740, ICD-10-D-Q87410, ICD-10-D-Q87418, ICD-10-D-Q8742, ICD-10-D-Q8743 | | | | | | |
|  |  |  |  |  |  |  |  |  |
|  | Ehlers-Danlos Syndrome | ICD-9-D-75983, ICD-10-D-Q796, ICD-10-D-Q7960, ICD-10-D-Q7961, ICD-10-D-Q7962, ICD-10-D-Q7963, ICD-10-D-Q7969} | | | | | | |
|  |  |  |  |  |  |  |  |  |
|  | Rheumatoid Arthritis | ICD-9-D-7140, ICD-10-D-M069, ICD-10-D-M0579, ICD-10-D-M0609, ICD-10-D-M059, ICD-10-D-M0600, ICD-10-D-M0689, ICD-10-D-M0589, ICD-10-D-M0540, ICD-10-D-M0800, ICD-10-D-M0570, ICD-10-D-M0680, ICD-10-D-M0580, ICD-10-D-M083, ICD-10-D-M0569, ICD-10-D-M0550, ICD-10-D-M0560, ICD-10-D-M06861, ICD-10-D-M05761, ICD-10-D-M06862, ICD-10-D-M06061, ICD-10-D-M0820, ICD-10-D-M05762, ICD-10-D-M06062, ICD-10-D-M0809, ICD-10-D-M0559, ICD-10-D-M05712 | | | | | | |
|  |  |  |  |  |  |  |  |  |
|  |  |  |  |  |  |  |  |  |
|  |  |  |  |  |  |  |  |  |
|  |  |  |  |  |  |  |  |  |
|  |  |  |  |  |  |  |  |  |
|  |  |  |  |  |  |  |  |  |
|  | SLE | ICD-9-D-7100, ICD-10-D-M329, ICD-10-D-M3210, ICD-10-D-M3219, ICD-10-D-M328, ICD-10-D-M3214, ICD-10-D-M320, ICD-10-D-M3213, ICD-10-D-M3212, ICD-10-D-M3215, ICD-10-D-M3211 | | | | | | |
|  |  |  |  |  |  |  |  |  |
|  |  |  |  |  |  |  |  |  |
|  | Sjogren Syndrome | ICD-10-D-M3500, ICD-10-D-M3501, ICD-10-D-M3502, ICD-10-D-M3503, ICD-10-D-M3504, ICD-10-D-M3509 | | | | | | |
|  |  |  |  |  |  |  |  |  |
|  | Dermatomyositis | ICD-9-D-7103, ICD-10-D-M3310, ICD-10-D-M3313, ICD-10-D-M3312, ICD-10-D-M3300, ICD-10-D-M3319, ICD-10-D-M3311, ICD-10-D-M3302, ICD-10-D-M3309, ICD-10-D-M3301, ICD-10-D-M3303 | | | | | | |
|  |  |  |  |  |  |  |  |  |
|  |  |  |  |  |  |  |  |  |
|  | Polymyositis | ICD-9-D-7104, ICD-10-D-M3320, ICD-10-D-M3321, ICD-10-D-M3322, ICD-10-D-M3329, ICD-10-D-M3390, ICD-10-D-M3391, ICD-10-D-M3392, ICD-10-D-M3393, ICD-10-D-M3399 | | | | | | |
|  |  |  |  |  |  |  |  |  |
|  |  |  |  |  |  |  |  |  |
|  | Mitochondrial Disease | ICD-9-D-27787, ICD-10-D-E8840, ICD-10-D-E8849, ICD-10-D-G713 | | | | | | |
|  | Cancer | ICD-9-D-1960:ICD-9-D-1999, ICD-10-D-C770:ICD-10-D-C809 | | | | | | |
